# The combined transarterial and transvenous onyx embolization of dural arteriovenous fistula of hypoglossal canal via the external jugular vein and facial vein: A case report

**DOI:** 10.3389/fsurg.2022.1043340

**Published:** 2023-01-06

**Authors:** Biao Yang, Yeqing Ren, Yongqiang Wu, Wenju Zhang, Yanqi Sun, Xiaolong Guo, Ming Lv, Geng Guo

**Affiliations:** ^1^ Department of Neurosurgery, The First Hospital, Shanxi Medical University, Taiyuan, China; ^2^ Department of Neurosurgery, Xuanwu Hospital, Capital Medical University, Beijing, China; ^3^ Department of Interventional Neuroradiology, Beijing Tian Tan Hospital, Capital Medical University, Beijing, China

**Keywords:** dural arteriovenous fistula, hypoglossal canal, anterior condylar confluence, transarterial embolization, transvenous embolization, external jugular vein

## Abstract

Dural arteriovenous fistulas of the hypoglossal canal (HCDAVFs) involving the anterior condylar confluence (ACC) and anterior condylar vein (ACV) are infrequent. Although transvenous embolization through the internal jugular vein (IJV) is the preferred treatment option for type I and II fistulas, it can be difficult if the IJV is unavailable. Here we report a rare case of HCDAVF in which the most common transvenous embolization access via IJV was not available. The patient underwent transarterial and transvenous onyx embolization. Transarterial embolization (TAE) aimed at controlling the arterial inflow and subsequently TVE was performed via the external jugular vein (EJV), the facial vein, the ophthalmic vein, the cavernous sinus, ACC, and ultimately to the fistula pouch. Complete obliteration of the HCDAVF was achieved without complications. We suggest that transvenous embolization (TVE) via the EJV and the facial vein can be effective in cases where trans-IJV is not possible.

## Introduction

Dural arteriovenous fistulas of the hypoglossal canal (HCDAVFs) are rare dural arteriovenous fistulas (DAVF) that involve anterior condylar confluence (ACC) and the anterior condylar vein (ACV) (1). HCDAVFs exhibit a rich venous network connecting with the ACV, lateral condylar vein, IJV, and vertebral venous plexus. They also present connections to the IPS, cavernous sinus, and the superior ophthalmic vein (2). Due to the complex vascular anatomical characteristics and various clinical manifestations, the diagnosis and treatment are challenging. Although the current primary option for treating HCDAVF types I and II is transvenous embolization (TVE) via internal jugular vein, the IJV may be unavailable in some situations, making treatment difficult (3).

In this study, the case of HCDAVF was diagnosed by digital subtraction angiography (DSA). The most common transvenous embolization access through the internal jugular vein was not available, and TVE was performed via the external jugular vein (EJV) and facial vein to occlude the fistula. The goal of transarterial embolization (TAE) before TVE was to control the arterial inflow. This case has advanced the understanding of HCDAVF and provided a new alternative approach when the most common internal jugular vein approach is not feasible.

### Case presentation

**Figure 1:**
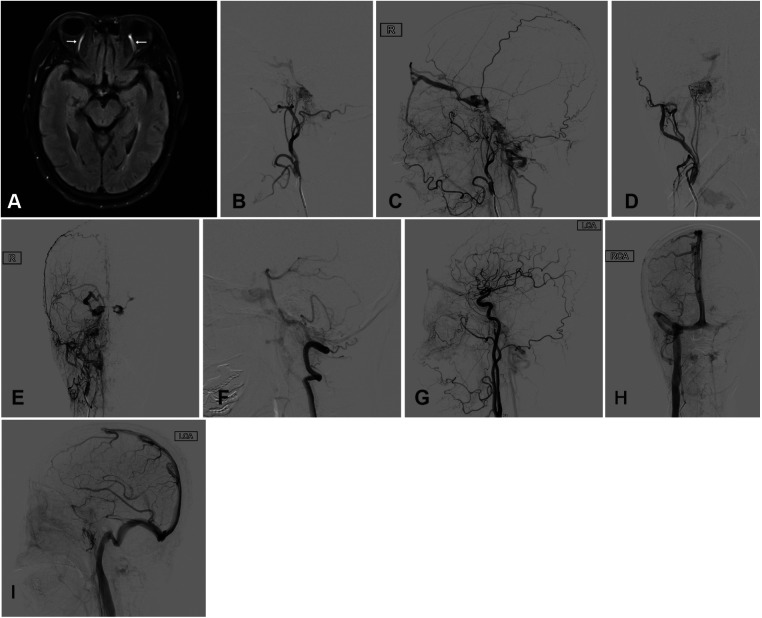
(**A**) MRI revealed mild dilation of bilateral superior ophthalmic veins (white arrows). Angiography of the right external carotid artery (**B–E**), right vertebral artery (**F**) and the left common carotid artery (**G**) showed the right HCDAVF, which was drained into the cavernous sinus through IPS vein, then entered the facial vein through the right ophthalmic vein, and finally reached the external jugular vein. HCDAVF, Dural arteriovenous fistulas of the hypoglossal canal. The patient's right internal jugular vein is not occluded (**H**) and the left IPS was occluded (**I**). IPS, inferior petrosal sinus.

A woman in her 70s presented with left eye pain and diplopia for 1 week. Examination revealed that the diameters of the bilateral pupils were unequal, with the left pupil measuring 4.0 mm and the right pupil 2.5 mm. She exhibited left-side ptosis, a slow pupillary light reflex, diplopia, bilateral congestion, and edema of the conjunctiva. Magnetic resonance imaging (MRI) revealed a slight dilation of bilateral superior ophthalmic veins. DSA showed a fistula in the right ACC. The fistula was supplied by the bilateral (mainly right) neuromeningeal trunk of ascending pharyngeal artery and the meningeal branches of the right vertebral artery. Retrograde drainage occurred exclusively via the right IPS toward the cavernous sinus. Then, it was drained through the right superior ophthalmic vein to the facial vein, and ultimately to the external jugular vein. It was also drained through the intercavernous sinus to the left cavernous sinus, the left ophthalmic vein, the left facial vein, and to the left external jugular vein. This patient had a bilateral inferior petrosal sinus occlusion with no good inferior petrosal sinus access (Figure 1).

The procedure was performed under endotracheal intubation anesthesia. The 5F arterial sheath and 6F venous sheath were placed through right transfemoral access. Heparin was given intravenously with a bolus dose of 50 U/kg. A 5F catheter was placed on the guide wire at the start of the right external carotid artery. A 6F guide catheter was placed at the start of the right external jugular vein.

**Figure 2:**
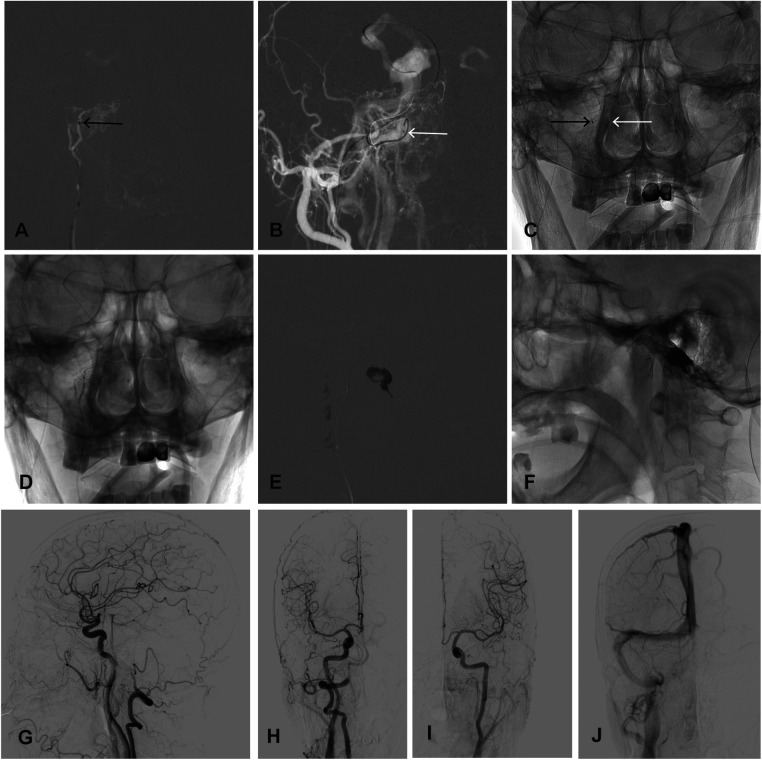
(**A**) The echelon-10 microcatheter (black arrow) was superselected for the right ascending pharyngeal artery. (**B**) The tip of the Marathon microcatheter (white arrow) was positioned at the right ACC. (**C**) The frontal view showed the position of the microcatheters. Onyx-18 was injected through the Echelon-10 microcatheter (**D**) and then injected into the venous bag through the Marathon microcatheter (**E**) and the shape of the venous bag (**F**) was obtained. The post-embolization angiography of the right innominate artery (**G, H**), left CCA (**I**) and late venous phase (**J**) showed complete obliteration of the fistula. ACC, anterior condylar confluence; IPS, inferior petrosal sinus.

Under the roadmaps, the superselective Echelon-10 microcatheter (Medtronic, Inc., USA) was routed through the 5F guide wire (Johnson, USA) to the right ascending pharyngeal artery (Figure 2A). A superselective angiogram via the microcather confirmed the location of venous pouch.

The most direct route to the ACC via the IJV was not available because the connection between the IJV and the ACC was occluded. The Marathon microcatheter (Medtronic, Inc., USA) over Synchro-10 microline (Stryker, USA) was advanced to the right IPS via the right external jugular vein, facial vein, ophthalmic vein, and cavernous sinus, and was superselected to the right anterior condylar confluence (Figures 2B,C).

The Echelon-10 microcatheter was rinsed with DMSO, and approximately 0.6 ml Onyx-18 (Medtronic, Inc., USA) was injected through the microcatheter to reduce the arterial inflow (Figure 2D). Onyx-18 was then slowly injected into the venous pouch through the Marathon microcatheter to obtain the shape of the venous pouch. The injection was stopped immediately when the reflux of Onyx-18 to the IPS was observed, and approximately 3 ml Onyx-18 was used (Figures 2E,F). The post-embolization angiography demonstrated complete obliteration of the fistula (Figures 2G–J).

### Outcome and follow-up

After 1 week of embolization, the ocular symptoms of the patient disappeared. No immediate or delayed1 complications occurred. There was no recurrence at half-year follow-up examinations. The patient did not complain of any discomfort symptoms and expressed great satisfaction with the operation results.

## Discussion

HCDAVF is a rare subtype of dural arteriovenous fistulas (DAVF) that accounts for less than 5% of the total intracranial DAVF (4, 5). Spittau et al. classified the dominant vein drainage patterns of HCDAVFs into three categories according to different clinical presentations (3). 62.5% of HCDAVFs with type 1 venous drainage to IJV and/or the VVP were associated with pulsatile tinnitus. 23.3% of HCDAVFs showed retrograde cavernous venous drainage and ophthalmic venous drainage (type 2), which were associated with orbital symptoms. 14.2% of HCDAVFs showed exclusive cortical or perimedullary venous drainage (type 3) with intracranial hemorrhage or cervical myelopathy. According to this classification, the HCDAVF in this case is presented with ptosis, diplopia, conjunctival hyperemia, and edema, and retrograde drainage exclusively via the right IPS toward the cavernous sinus; is classified as type 2.

The treatment of HCDAVF should be based on its location and anatomic characteristics. At present, the main treatment methods are TAE, TVE, surgery, and radiotherapy. TAE carries a significant risk of inferior cranial nerve palsy and embolic stroke because the neurovessels of the inferior cranial nerves (IX–XII) are derived from the neuromeningeal trunk of the ascending pharyngeal artery (6–8).

TVE is considered to be the main treatment for type I and II HCDAVFs with high safety and efficacy. According to Spittau et al. the clinical cure rate of TVE is 91%, and the permanent morbidity is 2.9%. The most common route is via the internal jugular vein (3, 4). TVE embolization materials include coils and liquid embolizers. In the previous treatment of HCDAVFs (9), Takemoto et al. used coils to control the fistula flow and provide secured anchoring to the onyx cast in order to avoid inappropriate migration of the onyx. Ye et al. described eight cases of HCDAVFs treated by intravenous balloon-assisted onyx embolization with anterograde venous drainage to the IJV. The intravenous balloon-assisted technique allowed onyx to penetrate better and prevented onyx from dispersing to the IJV (10). Besides, Crockett et al. reported that intraoperative selective cone-beam CT angiography (CBCTA) and intraoperative cone-beam CT (CBCT) were useful adjunct tools for planar DSA during the examination and intravenous coil embolization of HCDAVFs (4).

However, in some cases, trans-IJV access is not possible, because the connection between the IJV and the venous shunt is extremely tortuous or even closed. And then other alternative approaches are applied. Dahl et al. reported a case of successful treatment of HCDAVF by intravenous coil embolization through the deep cervical vein as an alternative access to the ACC after the failure of the trans-IJV approach (11). Diaz et al. reported a case of HCDAVF in which, after embolization and surgical failure, the anterior condylar vein was punctured percutaneously at the hypoglossal foramina and occluded with onyx after placing two anchor coils (6). Júnior et al. reported a case of HCDAVF in which the ACC was occluded by direct puncture of the cavernous sinus after transvenous access was unavailable (12). In our case, it was impossible for the most common intravenous embolization to pass the IJV, while the natural venous drainage of the external jugular vein and the facial vein might be feasible. We used a combination of transarterial and transvenous onyx embolization. TAE was used to control the arterial inflow, and subsequently, TVE was performed via the external jugular vein, facial vein, ophthalmic vein, cavernous sinus, subpetrosal sinus ACC, and ultimately to the fistula pouch to complete the obliteration of the fistula.

## Conclusions

In conclusion, we reported a rare case of HCDAVF, and the patient was successfully treated with combined transarterial and transvenous onyx embolization via the EJV and facial vein. Complete recovery of the patient was achieved. This case has advanced the understanding of HCDAVFs and provided a new alternative approach when the most common drainage (IPS to IJV) is not available.

## Data Availability

The original contributions presented in the study are included in the article/Supplementary Material, further inquiries can be directed to the corresponding authors.
